# Motion and Gender-Typing Features Interact in the Perception of Human Bodies

**DOI:** 10.3389/fnins.2020.00277

**Published:** 2020-04-21

**Authors:** Giulia D’Argenio, Alessandra Finisguerra, Cosimo Urgesi

**Affiliations:** ^1^PhD Program in Neural and Cognitive Sciences, Department of Life Sciences, University of Trieste, Trieste, Italy; ^2^Laboratory of Cognitive Neuroscience, Department of Languages and Literatures, Communication, Education and Society, University of Udine, Udine, Italy; ^3^Scientific Institute, IRCCS E. Medea, Pasian di Prato, Udine, Italy

**Keywords:** body image, sex categorization, gender perception, gender bias, attractiveness, body aesthetic, implied motion

## Abstract

The human body conveys socially relevant information, including a person’s gender. Several studies have shown that both shape and motion inform gender judgments of bodies. However, while body shape seems to influence more the judgment of female bodies, body motion seems to play a major role in the judgments of male bodies. Yet, the interdependence of morphologic and dynamic cues in shaping gender judgment and attractiveness evaluation in body perception is still unclear. In two experiments, we investigated how variations of implied motion and shape interact in perceptual and affective judgments of female and male bodies. In Experiment 1, participants were asked to provide ratings for masculinity and femininity of virtual renderings of human bodies with variable gender-typing features and implied motion. We found evidence of a tendency to perceive bodies in static poses as more feminine and bodies in dynamic poses as more masculine. In Experiment 2, participants rated the same pictures for dynamism and pleasantness. We found that male bodies were judged more dynamic than female bodies with the same pose. Also, female bodies were liked more in static than in dynamic poses. A mediation analysis allowed us to further shed light on the relationship between gender-typing features and motion, suggesting that the less is the movement conveyed by a female body, the greater is an observer’s sensitivity to its femininity, and this leads to a more positive evaluation of its pleasantness. Our findings hint to an association between stillness and femininity in body perception, which can stem from either the evolutionary meaning of sexual selection and/or the influence of cultural norms.

## Introduction

Gender is one of the most significant constructs in human social organization. Humans can rapidly differentiate between male and female conspecifics, relying on a heightened sensitivity to the biological commonalities that make an individual a male or a female. The evolution of this ability has been likely driven by the reproductive necessity to recognize the natural features of a potential mate. Even when primary sex characteristics are not visible, other morphological features of the body can easily mark sexual dimorphism. Among these features, the waist-to-hip ratio (WHR), which is the circumference of the waist relative to the hips, is considered an important body cue to accurately discriminate men from women (Lippa, 1983; Johnson, 2004; Johnson et al., 2012). Indeed, after puberty, women accumulate more fat on the hips than men and, over the years, the similarity in WHR between boys and girls decreases (Singh, 1993). Another sexually dimorphic feature is the shoulder-to-hip ratio (SHR), which is the circumference of the shoulders relative to the hips, which tends to be higher in men than in women (Hugill et al., 2011). Accordingly, eye-tracking studies have shown that, while men spend more time examining the waist area of women’s bodies, women focus their attention on the upper body of men (Dixson et al., 2011, 2014), suggesting that the use of WHR and SHR might be more important for evaluating female and male bodies, respectively.

However, not only the morphological appearance of the body, but also its dynamicity may inform about gender, particularly when morphological features provide ambiguous information, such as when people are seen heavily dressed and/or from a considerable physical distance (Johnson and Tassinary, 2005). Since men and women move differently, gender judgment is tightly linked to body movement perception (Kozlowski and Cutting, 1977; Mather and Murdoch, 1994; Kerrigan et al., 1998). Accordingly, people can accurately detect gender from mere point-light displays of walking figures (Kozlowski and Cutting, 1977; Mather and Murdoch, 1994). Indeed, while walking with more hip translations (sway) is judged as more feminine, walking with more shoulder translations (swagger) is judged as more masculine. Further, McDonnell et al. (2007) have demonstrated that motion *per se* can guide gender judgment when morphological cues are ambiguous, for example, in case of androgynous bodies. In fact, when typical feminine or masculine walking kinematics is applied to a neutral body figure, gender judgment follows the applied walking kinematics (McDonnell et al., 2007). Thus, there is evidence that people consider both morphologic and dynamic cues as reliable information on which they build their gender judgments.

Interestingly, the compatibility between morphological and dynamic cues of gender categorization leads to greater aesthetic appreciation of a body, with swaying female bodies and swaggering male bodies being judged more attractive (Johnson and Tassinary, 2007). From an evolutionary perspective, body attractiveness plays a key role in sexual selection, as it is the main vehicle to appeal a partner and prompt reproductive behavior. However, since the factors regulating health and reproductive capabilities cannot be directly observed, sexual selection may have favored psychological adaptations to attend to bodily features that are correlated with a greater procreative value (Symons and Buss, 1994; Sugiyama, 2004). Within this framework, studies have shown that different bodily features signal procreative value in men and women. For example, a man can increase his reproductive success by choosing a woman who is highly fecund, thus attending more to morphological body features (e.g., WHR) that signal female fertility. Indeed, WHR has been documented as a strong indicator of female fertility (Singh, 1993) as well as a reliable measure in the judgment of women’s body attractiveness (Grammer et al., 2003; Singh and Singh, 2011) in a consistent way across many populations in men’s preferences (Dixon et al., 2007; Sorokowski and Sorokowska, 2012; Brooks et al., 2015). Conversely, women may be more prone to choose a male mate with greater competitive drive (Archer, 2009) in order to provide resources to raise her offspring. This is consistent with data showing that women rate as more attractive taller and more muscular men (Mautz et al., 2013) and that body composition in men is not related to sperm motility, an indicator of male fertility (Fejes et al., 2005), but rather with physical strength (Windhager et al., 2011). Further, women seem to infer the health and strength quality of a man via active displays of the body, such as in dance (Hugill et al., 2009, 2010; Neave et al., 2011), which can be viewed as an important part of male courtship (Sheets-Johnstone, 2005). This is in keeping with the special role of body movements in communicating men’s formidability (i.e., fighting ability and resource-holding potential).

The pressure of sexual selection on different survival values of men and women has blended into stereotypical expectations about gender-specific features that men and women are encouraged to exhibit in given socio-cultural contexts. Indeed, society not only shapes personality and behavior, but also the way in which the body appears (Nicholson, 1994). Applying undeniable societal pressure toward a thin-ideal shape for girls (Blaivas et al., 2002; Hawkins et al., 2004; Grabe et al., 2008) and an increased muscular body for boys (Hawkins et al., 2004; Daniel and Bridges, 2010), mass media reinforce the embodiment of gender-role norms. For example, it has been documented that men use physical activity to showcase their masculinity, since it helps to emphasize muscularity and, consequently, to be identified as a stronger individual (Drummond, 2008). Thus, although within the last decades women have challenged the myth that sport is a prerogative of men, the overrepresentation of male athletes in the media compared to female athletes is still persistent, with over 94% of coverage being dedicated to men (Cooky et al., 2015; Hovden and von der Lippe, 2019). Furthermore, the sports achievements of male athletes are regarded as more important than those of female athletes, who are rather mentioned for their physical attractiveness. In this sense, while morphological cues are emphasized for the judgment of women (Tolman et al., 2006), masculine gender-typing features are more related to performance and activities, including personal attributes like being a powerful, strong, and efficacious individual (Mishkind et al., 1986; McCreary et al., 2005).

In sum, both evolutionist and socio-cultural studies have provided numerous clues about the association between specific forms and movements of a body and the perception of its femininity/masculinity. Furthermore, these studies have also shown that morphological and dynamic cues may differently influence the aesthetic appreciation of a male or female body. What is unclear, however, is whether and how form and movement cues may influence each other. A previous study (Cazzato et al., 2012) showed not only that thinner and more dynamic bodies received more positive aesthetic appreciation, but also that the perception of the size of a body was influenced by its dynamicity, with the same body being judged thinner when displayed in a dynamic than in a static posture. Here, we investigated how variations of gender-typing morphological features (e.g., WHR) and dynamicity (static vs. moving posture) influence each other in guiding gender judgment and aesthetic appreciation of a body. To this aim, we created a pool of images of 3-D rendered bodies differing for the multivariate embodiment of sex-specific morphological features and for implied movement. In two experiments, participants were asked to rate them for femininity and masculinity (Experiment 1) and for dynamism and attractiveness (Experiment 2). We predicted that static and dynamic body postures should differently influence the perception of the gender-typing features of male and female bodies. In particular, we expected that static poses applied to a morphologically female figure would increase the perception of its femininity as well as the appreciation of its aesthetic value; conversely, dynamic poses would lead to the same pattern of effects in the case of a morphologically male figure.

## Experiment 1

### Materials and Methods

#### Participants

A sample of 30 students (17 female) from the University of Udine (Italy) took part in the experiment in return for course credits. They were aged 18–35 years (mean = 26.63, *SD* = 5.15) and reported normal or corrected-to-normal visual acuity in both eyes. No participants reported any current neurological or psychiatric disorders. Written informed consent was obtained from each participant. The study procedures were approved by the institutional ethics review board (Commissione di Garanzia per il rispetto dei principi etici nell’attività di ricerca sugli esseri umani, Department of Language and Literature, Communication, Education and Society, University of Udine, Italy; Study Protocol CGPER-2019-12-09-02) and were in accordance with the ethical standards of the 1964 Declaration of Helsinki. Participants were naive to the aims and hypothesis of the experiment and a study debriefing was conducted at the end of the experiment. All participants were right-handed as ascertained with standard handedness inventory (Oldfield, 1971).

#### Stimuli

**FIGURE 1:**
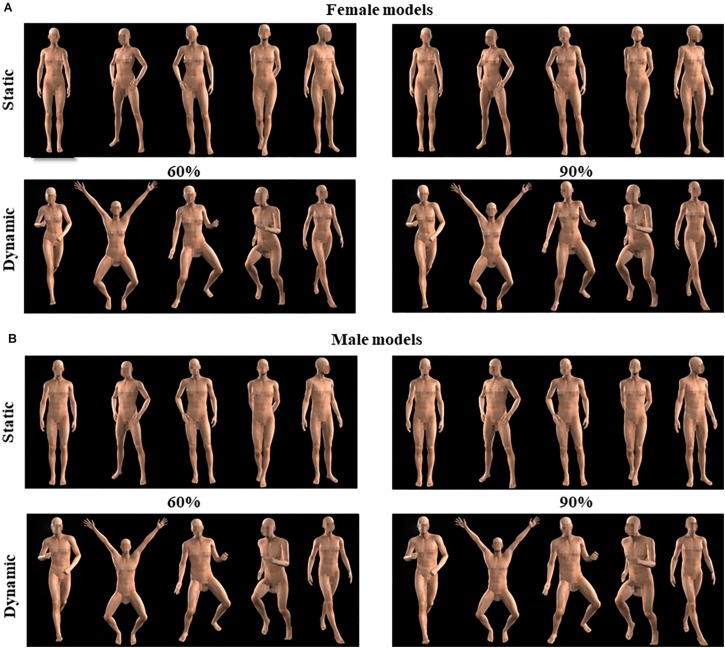
Examples of female and male virtual models used in the study. The examples depict the body sex-typing variation of either static or dynamic female **(A)** and male **(B)** models.

To systematically control for the masculinity/femininity traits and implied motion of our body stimuli, we used the software Character Creator 3.0 (Reallusion, San Francisco, CA, United States). Four virtual-human models (two female and two male models) were previously selected from the default database. By using the software function to manipulate the percentage of masculinity/femininity traits embodied by a neutral body, we produced two different versions of each model setting the amount of gender typicality traits at 60 or 90%. This allowed us to obtain more or less masculine/feminine bodies for each identity. Moreover, each body was rendered in 10 different daily poses, namely, five static (e.g., standing, open, idle, and turned postures) and five moving poses (e.g., running, walking, jumping, dancing, moving), selected from the default folders of static and dynamic poses available in Character Creator (see Figure 1). Bodies could be viewed from a frontal or three-quarter view and were pictured against a black background. Thus, in total, we had two female and two male models depicted in two different versions (60 or 90% gender typicality) and rendered in 10 different postures for a total of 80 different body images. Furthermore, pictures were imported into GIMP 2.10.8 (GNU Image Manipulation Program, Berkeley, CA, United States) in order to produce a mirrored version of each image and thus obtain a pool of 160 different body stimuli. Importantly, for all images, the head, pectoral, and pelvic areas were blurred in order to mask primary sexual characteristics, keeping, however, enough morphological information to visually convey the sexual phenotype (see Figure 1).

#### Procedure

The experiment was created with E-Prime software (version 2.0, Psychology Software Tools, Inc., Pittsburgh, PA, United States). During the experimental session, participants sat 60 cm away from a 19-in PC monitor (resolution: 1,360 × 768 pixels; refresh frequency: 60 Hz), on which 600 × 600 pixel images were presented one at a time at the center of the screen. In different blocks, participants were asked to provide two different judgments about the bodies, namely, either a femininity judgment (“How much do you think this body is feminine?”; in Italian, “*Quanto ritieni che questo corpo sia femminile?”*) or a masculinity judgment (“How much do you think this body is masculine?”; in Italian, “*Quanto ritieni che questo corpo sia maschile?”*). Each trial started with the appearance of the body image with a 1–7 Likert-scale below, which remained on the screen until response. Participants rated the two attributes for each body by using 1 (not at all) to 7 (very much) keyboard keys with both hands. Soon after participants’ response, the image disappeared and the next trial was presented. The same stimuli were randomly presented once in the Femininity block and once in the Masculinity block. The order of presentation of each block was counterbalanced across participants.

#### Data Handling

All the analyses were performed using ANOVA designs implemented in STATISTICA software (Stat Soft, version 10, StatSoft Inc., Tulsa, OK, United States). For each experimental block (i.e., Femininity and Masculinity), individual rating values were collected and separately submitted to three-way ANOVA with Posture (static vs. dynamic poses), Gender (male vs. female stimuli), and Typicality (60% vs. 90% gender traits) as within-subject variables and Gender group (male vs. female observers) as a between-subjects variable. Significant interactions were explored with the Tukey *post-hoc* test to correct for multiple comparisons. Significance threshold was set at *p* < 0.05. Effect size was estimated with partial eta squared ($\eta_{p}^{2}$). Judgment values are shown as mean ± standard error (SE).

### Results

#### Femininity Judgments

**FIGURE 2:**
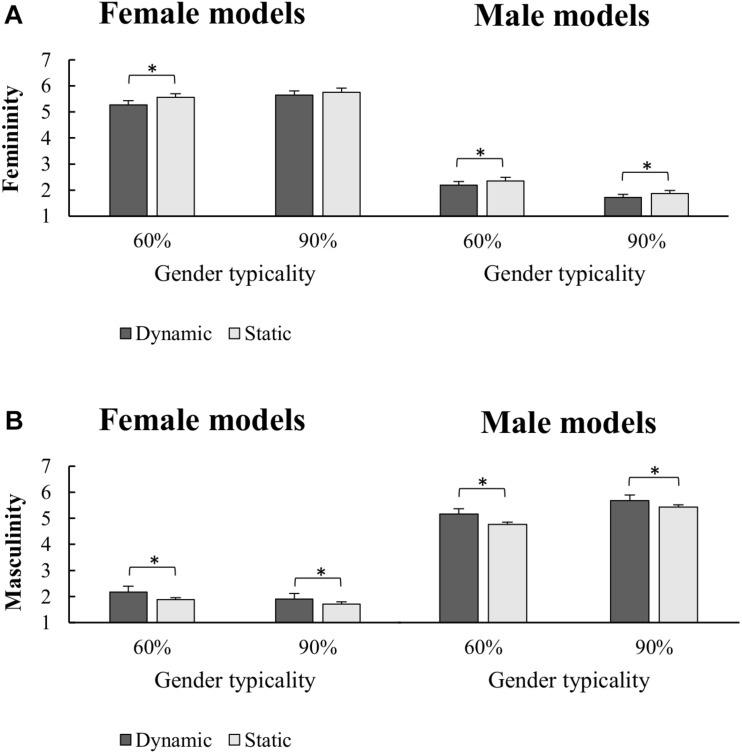
Mean and standard error of mean for Femininity **(A)** and Masculinity **(B)** judgments of Female and Male models as a function of implied motion (static/dynamic) along the two categories of sex-typing traits expressed (60%/90%). Asteriks indicate significant pairwise comparisons.

The ANOVA on femininity judgments (Figure 2A) showed, as expected, significant main effects of Gender [*F*_(1,29)_ = 326.98; *p* < 0.001; $\eta_{p}^{2}$ = 0.919] and Typicality [*F*_(1,29)_ = 5.68; *p* = 0.024; $\eta_{p}^{2}$ = 0.164], which were further qualified by a significant Gender × Typicality interaction [*F*_(1,29)_ = 134.17; *p* < 0.001; $\eta_{p}^{2}$ = 0.822]. Tukey *post-hoc* tests [mean square error (MSE) = 0.06525, *df* = 29] showed that femininity judgments continuously increased from the 90% male stimuli, which were judged as the least feminine bodies, to the 90% female stimuli, which were judged as the most feminine bodies (all *p*s < 0.001). Importantly, however, we also found a main effect of Posture [*F*_(1,29)_ = 10.67; *p* = 0.003; $\eta_{p}^{2}$ = 0.2690], showing higher level of femininity for static than dynamic poses. Finally, the Posture × Gender × Typicality interaction [*F*_(1,29)_ = 4.587; *p* = 0.04; $\eta_{p}^{2}$ = 0.1365] was also significant, showing that the effect of posture was different for body figures with different feminine/masculine typicality. Tukey *post-hoc* comparisons (MSE = 0.02869; *df* = 29) revealed that both 60% (*p* = 0.017) and 90% (*p* = 0.019) male stimuli received higher femininity judgments when displayed in static than dynamic poses. The same effect of posture, however, was only obtained for the 60% (*p* < 0.001), but not the 90% (*p* = 0.525) female stimuli. Furthermore, femininity judgments increased with higher feminine typicality and with lower masculine typicality, independently from postures (all *p*s < 0.001). However, the 60% static female stimuli received comparable feminine judgments than the 90% dynamic ones (*p* = 0.289), suggesting that a static posture increased the feminine judgments of a low-typical body up to the level of a dynamic typical female body (or that a dynamic posture reduced the feminine judgments of a typical female body to the level of a static low-typical body). No significant main effect or interaction of Gender group was obtained but a Gender group × Gender interaction [*F*_(1,28)_ = 6.053; *p* = 0.02; $\eta_{p}^{2}$ = 0.1777], which showed that the difference between female and male models tended to be higher for female than male participants; however, *post-hoc* test did not reveal any significant between-group difference (all *p*s > 0.27).

#### Masculinity Judgments

The ANOVA on masculinity judgments (Figure 2B) showed significant main effects of Gender [*F*_(1,29)_ = 303.36; *p* < 0.001; $\eta_{p}^{2}$ = 0.913] and Typicality [*F*_(1,29)_ = 26.338; *p* < 0.001; $\eta_{p}^{2}$ = 0.476] as well as a significant two-way Gender × Typicality interaction [*F*_(1,29)_ = 171.994; *p* < 0.001; $\eta_{p}^{2}$ = 0.856]. Tukey *post-hoc* tests [MSE = 0.0592, *df* = 29] showed a specular pattern of results as compared to femininity judgments, with continuously increasing masculinity judgments from the 90% female stimuli, which were judged as the least masculine bodies, to the 90% male stimuli, which were judged as the most masculine bodies (all *p*s < 0.001). Again, we found a main effect of Posture [*F*_(1,29)_ = 32.85; *p* < 0.001; $\eta_{p}^{2}$ = 0.531], which revealed higher judgments of masculinity for dynamic than static poses and was qualified by a significant Posture × Typicality interaction [*F*_(1,29)_ = 8.74; *p* = 0.006; $\eta_{p}^{2}$ = 0.232]. Tukey *post-hoc* tests [MSE = 0.02808, *df* = 29] showed that dynamic poses led to higher masculine judgments independently from gender typicality, even if the effect of posture was higher for the 60% (dynamic vs. static pose difference, 0.694 ± 0.115) than for the 90% (dynamic vs. static pose difference, 0.439 ± 0.1) bodies [planned comparison, *F*_(1,29)_ = 8.74, *p* = 0.006]. No significant main effect or interaction of Gender group was obtained (all *F* < 2.928, *p*s > 0.1).

### Discussion

The results of Experiment 1 showed that implied motion modulated the perception of feminine gender-typing morphological features, since the same low-typical bodies received lower feminine and higher masculine judgments when displayed in dynamic than in static poses. This points to an association between stillness and femininity. Importantly, the effects of implied motion on femininity and masculinity judgments were comparable for male and female observers, since the effect of posture was not modulated by gender group. From this pattern of results, however, it is not possible to discern whether gender-typing feminine forms may also modulate the perception of implied motion conveyed by a static picture of a body. Furthermore, it is also unclear whether the compatibility between two seemingly associated body cues, namely, stillness and femininity, also affects body aesthetic appreciation, as shown for gender-typing bodies moving in a gender-typical way (Johnson and Tassinary, 2007). To address these issues, we implemented a second experiment testing a subset of participants who took part to Experiment 1 and agreed to complete a second session.

## Experiment 2

### Materials and Methods

The same stimuli, procedure, and data handling approach as in Experiment 1 were used, but participants (*N* = 21, 11 women) aged 19–34 years (mean = 26.75, *SD* = 4.82) were asked to judge, in separate blocks, the dynamism (“How much do you think this body is dynamic?”; in Italian, “*Quanto ritieni che questo corpo sia dinamico?*”) or the pleasantness (“How much do you like this body?”; in Italian, “*Quanto ti piace questo corpo?*”) of the stimuli. The order of block presentation was balanced across participants. The same repeated-measure variables (Posture × Gender × Typicality) as in Experiment 1 were tested, but the Gender group was not tested since it did not modulate the effects of implied motion on masculinity/femininity judgments in Experiment 1 in spite of greater sample size.

### Results

#### Dynamism Judgments

**FIGURE 3:**
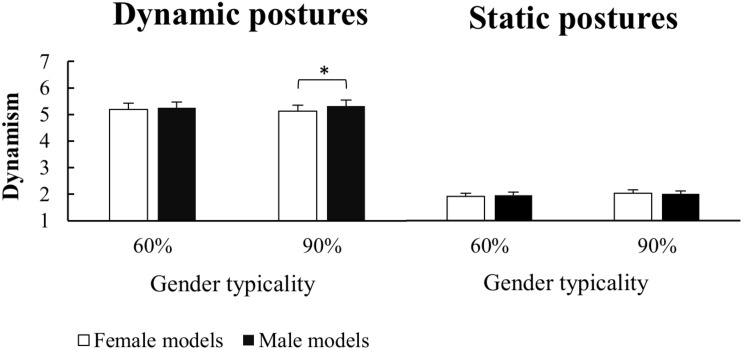
Mean and standard error of mean for Dynamism judgment of Dynamic and Static postures as a function of gender (female/male) along the two categories of sex-typing traits expressed (60%/90%). Asteriks indicate significant pairwise comparisons.

The three-way Posture × Gender × Typicality repeated-measures ANOVA on dynamism judgments (Figure 3) showed a significant main effect of Posture [*F*_(1,20)_ = 211.22; *p* < 0.001; $\eta_{p}^{2}$ = 0.914], with higher dynamism judgments for dynamic than static poses. Interestingly, the ANOVA also revealed a significant main effect of Gender [*F*_(1,20)_ = 6.16; *p* = 0.02; $\eta_{p}^{2}$ = 0.236], demonstrating that female stimuli were judged as less dynamic than the male ones, even if displaying the same poses. However, a significant Posture × Gender × Typicality interaction [*F*_(1,19)_ = 7.51; *p* = 0.01; $\eta_{p}^{2}$ = 0.283] indicated that the dynamism judgments of female and male stimuli were modulated not only by the displayed body posture but also by gender typicality. Tukey *post-hoc* comparisons [MSE = 0.01886; *df* = 20] showed that female bodies were judged less dynamic than male bodies only when they were displayed in a dynamic posture and with a 90% gender typicality (*p* = 0.005); conversely, no between-gender differences were obtained for the other figure types (all *p*s > 0.84). Furthermore, dynamic poses received higher dynamism judgments than static poses for all stimuli (all *p*s < 0.001), while for either male or female models, the 60% figures received comparable dynamism judgments to the 90% ones (all *p*s > 0.45).

#### Liking Judgments

**FIGURE 4:**
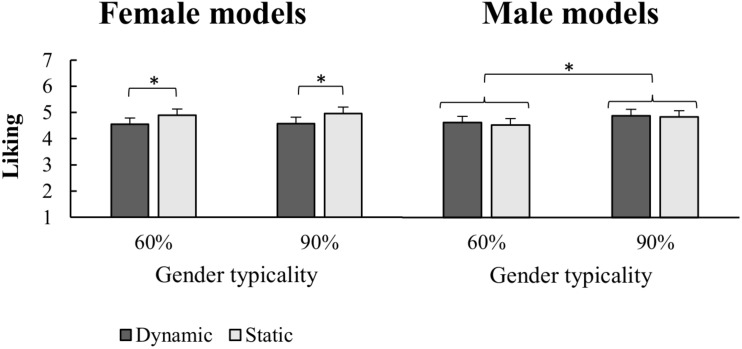
Mean and standard error of mean for Liking judgment of Female and Male models as a function of implied motion (static/dynamic) along the two categories of sex-typing traits expressed (60%/90%). Asteriks indicate significant pairwise comparisons.

The ANOVA on the aesthetic judgment values (Figure 4) showed a significant main effect of Typicality [*F*_(1,20)_ = 8.55; *p* = 0.008; $\eta_{p}^{2}$ = 0.299], further qualified by a Gender × Typicality interaction [*F*_(1,20)_ = 10.74; *p* = 0.004; $\eta_{p}^{2}$ = 0.349]. Tukey *post-hoc* test [MSE = 0.05433; *df* = 20] revealed that 90% male stimuli were judged more pleasant than the 60% ones (*p* < 0.001), while no typicality modulation of the liking judgments was obtained for the female stimuli (*p* = 0.822). Furthermore, less typical (i.e., 60%) female stimuli were liked more than less typical male stimuli (*p* = 0.026), while no between-gender difference was found for the 90% typicality stimuli (*p* = 0.444). Importantly, we found a significant Posture × Gender interaction [*F*_(1,20)_ = 9.59; *p* = 0.006; $\eta_{p}^{2}$ = 0.324], showing that also posture modulated the liking judgments of male and female bodies. Tukey *post-hoc* test comparisons [MSE = 0.020789; *df* = 20] indicated that female models were judged more pleasant in static than dynamic poses (*p* = 0.002), while no significant difference between static and dynamic poses for male models was found (*p* = 0.50).

#### Item Analysis

To further explore the relationship between gender typicality, dynamism and aesthetic appreciation, we switched the data from the participant into the stimulus space, averaging for each of the 180 stimuli the dynamism, liking, or gender judgments across the 21 participants who took part to both Experiments 1 and 2. Female and male stimuli were analyzed separately, considering the femininity judgments for female stimuli and masculinity judgments for male stimuli. A series of bivariate correlation analyses was performed within the two sets of stimuli. Within the female stimuli, the bivariate correlation analysis showed a significant positive correlation between Femininity and Liking judgments [*r*(80) = 0.58, *p* < 0.001], but a negative correlation between Dynamism and Liking judgments [*r*(80) = −0.35, *p* = 0.002]; furthermore, a negative correlation between Femininity and Dynamism judgments was also found [*r*(80) = −0.22, *p* = 0.047]. Within the male stimuli, results showed a significant positive correlation of Masculinity judgments with either Dynamism [*r*(80) = 0.23, *p* = 0.04] or Liking [*r*(80) = 0.65, *p* < 0.001] judgments, while dynamism did not significantly correlate with the liking of male bodies [*r*(80) = −0.072, *p* = 0.527]. In sum, the correlation analysis revealed that, for both male and female figures, greater gender typicality was associated with greater liking judgments. However, while dynamism was associated with greater masculinity judgments for male stimuli, more dynamic female stimuli were judged less feminine and less pleasant.

**FIGURE 5:**
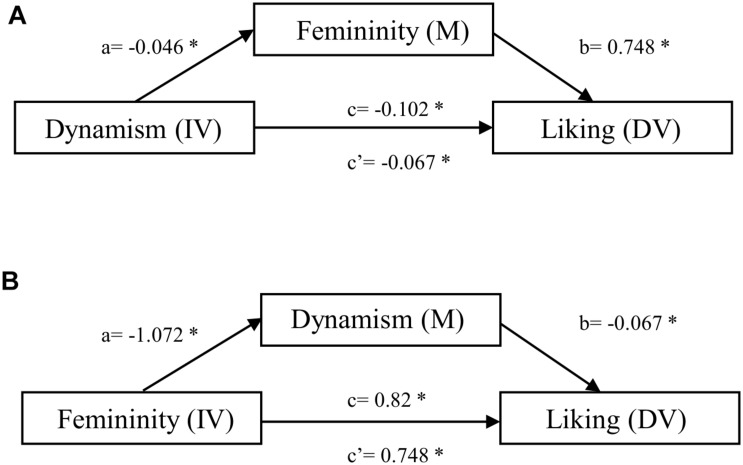
Path diagrams illustrating the two mediation models. The first model (**A**, upper panel) tested whether Femininity could mediate the influence of Dynamism (IV) on Liking judgments. The second model (**B**, lower panel) tested whether Dynamism could mediate the influence of Femininity (IV) on Liking judgments. For each path, values correspond to the unstandardized path coefficients for the association between variables. Namely, path a represents the relationship between IV and M, and path b represents the relationship between M and DV (controlling for IV). Path c represents the direct effect between IV and DV. Lastly, the indirect effect of the mediator, path c′, was quantified as the difference between the unstandardized path coefficients of the direct effect between the independent and the DV (path c), and the product of the unstandardized path coefficients, a and b. Asterisks denote significant (*p* < 0.05) regression coefficient. Significant differences between the direct and the indirect effects resulted from Sobel test in model **(A)**, indicating that the negative relationship between Dynamism and Liking judgment is mediated by Femininity. Asteriks indicate significant pairwise comparisons.

Considering the trine reciprocal correlation between femininity, dynamism, and aesthetic appreciation of female bodies, we conducted a mediation analysis in order to assess the relative role of femininity or dynamism in mediating the influence of the other variable on liking judgments of female stimuli. Thus, we used established methods of mediation analyses to understand whether the effect of the independent variable (IV) on the dependent variable (DV) could be explained by a mediator (M) (MacKinnon et al., 1995). In particular, in two separate models, we tested whether the femininity or the dynamism could mediate the influence on liking judgment, exerted respectively by the dynamism or the femininity variables (Figure 5). Mediation effects were tested using the Sobel test by applying the Goodman correction (Goodman, 1960; MacKinnon et al., 1995). One-tailed effects were tested since the direction of the mediation was predicted. In the first model (A), we speculated that the level of Femininity (M) could mediate the impact of the Dynamism (IV) expressed by a female body on its Liking (DV). Inserting Femininity as mediator, the model provided evidence for an indirect effect of Dynamism on Liking judgments, since the negative relation between Dynamism and Liking was significantly affected by the inclusion of Femininity as a mediator (*z* = −1.91, *p* = 0.05). Conversely, no evidence of mediation was obtained (*z* = 1.65, *p* = 0.1) in a second model (B), considering that the Dynamism (M) conveyed by a body could mediate the effect of Femininity (IV) and on its Liking (VD). Thus, mediation analyses suggested that more static female bodies were judged more feminine, leading to a more positive aesthetic appreciation.

### Discussion

The aim of Experiment 2 was to investigate how gender typicality influences the perception of implied motion and how these two body cues interact in shaping the aesthetic appreciation of a human body. For this reason, participants rated male and female bodies varying in gender typicality for their level of dynamism and pleasantness. The results showed that gender typicality influences the perception of motion implied by a body posture. In particular, female bodies with 90% gender-typing morphological features were judged as less dynamic than male bodies even if they had the same postures. Notably, this gender asymmetry was not found for the low-typical (i.e., 60% typicality) body figures, thus demonstrating the crucial impact of the salience of sex-specific traits in perceiving the motion evoked by body postures. This further strengthens the association between stillness and femininity. Furthermore, Experiment 2 also showed that the compatibility between these morphological and motion cues to the perception of body gender influenced the aesthetic appreciation of bodies. Indeed, in general, the more typical, 90% models were liked more than the less typical, 60% bodies, confirming that typicality of body forms is associated with greater pleasantness (Johnson and Tassinary, 2007). This effect, however, appeared stronger when people were asked to judge a male model, indicating that the salience of gender-typing attributes may be more important in the appreciation of male beauty. Importantly, we also found that female models in static poses were judged more pleasant with respect to female models in dynamic poses. Since the pleasantness of a body increases with its capability to express gender-specific features (Johnson and Tassinary, 2007), the higher rate of pleasantness for static female bodies seems to be consistent with the idea that stillness could enhance femininity appearance. This was also corroborated by an item-level mediation analysis showing that more static female bodies were judged more feminine and this led to a more positive aesthetic appreciation.

## General Discussion

The present study aimed to investigate how the manipulation of gender-specific morphological features and implied motion of a body interact in its judgments. To this end, we asked participants to rate the masculinity and femininity (Experiment 1) or the dynamism and pleasantness (Experiment 2) of a series of pictures depicting male and female bodies expressing different amounts of gender-typing features (60% vs. 90% typicality) and displayed in static or dynamic postures. As expected, participants assigned higher value of masculinity and femininity to more gender-typical male and female bodies, respectively. However, the most interesting finding was that also implied motion influenced the gender judgment of body figures, at least when they were displayed with less gender-typing features (i.e., 60% typicality). Indeed, participants tended to perceive low-typical female bodies as more feminine when displayed in static than dynamic poses and to perceive low-typical male bodies as more masculine in dynamic than static poses. Crucially, however, not only implied motion influenced the perception of the gender-typing features of a body figure, but also gender typicality influenced the perception of motion conveyed by a body posture. Indeed, we found that models with typical female-typing features were evaluated as less dynamic than models with typical male-typing features, even when they displayed the same pose. This pattern of results suggests that gender-typing morphological cues and implied motion interact in shaping the perception of body gender. When morphological cues are not clear, the perception of static or dynamic postures pushes gender perception toward a female or male body, respectively. In a similar vein, when the motion conveyed by a body is fuzzy (e.g., implied motion in body pictures), the perception of female- or male-typing features pushes motion perception toward stillness or dynamism, respectively.

Importantly, we also found, at both subject- and item-level analyses, that the association between stillness and femininity influenced the aesthetic appreciation of a body. Indeed, bodies with more gender-typing features (i.e., 90% typicality) were liked more than less-typical bodies (60% typicality). This is in line with the notion that the stereotypical representation of the body according to its gender has implications for its aesthetic appreciation (McCreary et al., 2005), reflecting a correlation between gender-typing features and the impression of a good-looking body (Johnstone, 1994; Grammer et al., 2003; Singh and Singh, 2011). However, we also found that, within female figures, the models in static poses were evaluated as more pleasant than those in dynamic poses. This may seem in contrast with studies showing that more dynamic dance poses are liked more (Calvo-Merino et al., 2008; Cross and Ticini, 2012; Kirsch et al., 2016) and that implied motion enhances the aesthetic appreciation of human bodies (Cazzato et al., 2012), in terms of either attribution of intrinsic perceptual properties to the stimulus (i.e., beauty) or observer’s attitude to it (i.e., liking or attractiveness). However, albeit gender-typical features were less salient in these previous studies as compared to our study, implied motion was found to be a better predictor of the aesthetic appreciation of male than female bodies (Cazzato et al., 2012). In addition, the different impact of static and dynamic stimuli in the judgment of female physical attractiveness has already been reported in adult actresses, showing that more feminine WHRs and larger breasts are considered desirable traits in static photographs whereas more androgynous body shapes are considered appropriate in stars that perform in movies (Voracek and Fisher, 2006). Here we found that static postures increased the aesthetic appreciation of female bodies. This effect could be due to a direct negative effect of implied motion on the appreciation of female attractiveness or be indirectly mediated by a masking of female-typical physical traits. However, the item mediation analysis allowed us to better delineate the relationship between femininity perception, stillness and aesthetic appreciation. In particular, we tested two models, based on the hypothesis that either stillness increased the perceived femininity of a female body and thus increased its pleasantness (Model A) or that femininity reduced the implied motion of a female body and thus reduced its pleasantness (Model B). The results provided evidence in favor of the first model, since perception of femininity was a key mediator of the negative relation between implied motion and liking. In other words, the effect of implied motion on the liking judgments of female bodies was better explained by an indirect effect mediated by femininity than by a direct effect of implied motion on liking. This supports the claim that stillness increased the aesthetic appreciation of a female body at least partially because it increased its gender typicality, likely facilitating the perception of feminine-typing features. In sum, our data suggest that femininity and stillness, on one hand, and masculinity and dynamism, on the other hand, are associated features in body representation, confirming clues from both sexual-selection and socio-cultural frameworks.

In a sexual-selection evolutionist framework, perceiving a static female body vs. a dynamic male body may boost the salience of gender-typing physical traits, such as WHR for women and muscularity for men. Numerous studies, indeed, have shown that a female body is strongly defined by the WHR, since it appears to be related to objective gender-specific qualities such as the levels of sex hormones (e.g., estradiol; De Ridder et al., 1990; Mondragón-Ceballos et al., 2015), the accessibility to fat resources suitable for fetal neurodevelopment (Lassek and Gaulin, 2008), and the more general capacity to sustain pregnancy (Singh, 1993). Obviously, WHR might only serve as a proxy for covariating bodily traits that shape the entire body phenotype and co-determine the judgment of body attractiveness (Brooks et al., 2015). Certainly, being able to select these qualities on the basis of visual cues increases the reproductive success of the species and, in this respect, the body shape of a woman could be considered as the best way to rapidly infer her femininity, meant as a set of biologically determined attributes. Since WHR is based on the computation of the waist and hip proportions, it is plausible that movements may affect its estimation altering shape and size perception. A body in motion, indeed, can provide misleading information about shape, for instance by producing overlaps of body parts (i.e., arms that cover hips while running). As shown in a recent eye-tracking study (Pazhoohi et al., 2020), WHR is widely view-dependent and movement pattern can cause variation in WHR detection, even if body proportions remain constant. On this view, dynamism may hinder the expression of the femininity of a woman by obscuring her salient shapes as compared to when staying in canonical static poses.

Conversely, as in many animal species, humans show sex differences in body composition and the amount of muscle mass appears to be greater in men than in women (Wells, 2007). Performing actions may accentuate the perception of body muscularity, thus biasing gender perception toward masculinity. Furthermore, male individuals seem to tend to disclose their masculinity right through movements (Darwin, 1871), as demonstrated by males of some species which use dance as a signal of neuromuscular condition (Maynard Smith, 1956) or flight ability (Williams, 2001). In humans, for example, it has been shown that men’s bodily symmetry, a measure that reflects the developmental stability of an organism (Moller and Swaddle, 1997; Polak, 2003) and preservation from morbidity and mortality (Stevenson, 2000), strongly correlates with their dance ability (Brown et al., 2005) and running performance (Manning and Pickup, 1998). This suggests that movements, rather than shape, may be a better predictor of men’s functional effectiveness.

As a legacy of sexual selection, the stereotypical association between femininity/stillness and masculinity/dynamism is reflected in socio-cultural norms, grounded on how people think men and women should differ. A domain in which this distinction is quite tangible is represented by sports context. Indeed, studies have suggested that, in most of Western countries, girls and women are less encouraged to participate in sports than boys and men (Eccles and Harold, 1991; Hartmann-Tews and Pfister, 2003) and, even in physical activities where women are predominant, such as performing arts (i.e., ballet), performance seems to be judged more on the basis of aesthetic features than body capability (Klomsten et al., 2005). Nevertheless, media images in sports endorses the stereotyped view of men’s and women’s bodies, emphasizing strength and physical abilities in the case of male athletes but featuring female performers in terms of a sexualized body (Von Der Lippe, 2002). This is in line with the present finding that perception of femininity appears to be intensified by a static body pose. In this regard, studies about “woman objectification,” which refers to the tendency to perceive a woman worth in light of her body appearance and sexual function, have demonstrated that the identification of the female body as an object available for satisfying the needs of men may diminish her attribution of agency (Cikara et al., 2011) and, consequently, underline her passive condition. Interestingly, recent researches have shown that images of female bodies are processed as a recollection of body parts rather than a whole figure (Bernard et al., 2012, 2015), a fragmentary process that is generally observed in the recognition of objects; notably, this pattern of visual perception occurs independently from the gender of the observer, demonstrating that such objectification of the female body involves women themselves. Thus, the well-proved association between femininity and object-related features could easily explain why static postures make bodies to appear more feminine. At the same time, men are encouraged to display their sex-typing features in keeping with contemporary masculine norms, which consider increased muscle mass as more masculine (Mishkind et al., 1986; McCreary et al., 2005). This may explain why men tend to express their gendered body through exercising and practicing physical activity. Accordingly, a study aimed at exploring the association between levels of exercise and patterns of masculinity in men undergoing androgen deprivation therapy has recently revealed that men who are aerobically active have higher levels of self-reported masculinity than those who are inactive (Langelier et al., 2018), highlighting the intersection of masculinity and physical activity. Further, women also seem to judge masculinity through body movements, since they assess a man’s physical strength and attractiveness on the basis of his gait (Fink et al., 2016).

The conclusions that can be drawn from this study need to be weighted in the light of important limitations. First of all, we investigated the effects of dynamic cues in body perception by using static pictures of bodies with implied motion. This allowed controlling for the amount of body views offered in videos of a moving or still person, but obviously limits the salience and naturalness of body movements. Nevertheless, there is evidence for common neurocognitive representation of actual and implied body movements (Urgesi et al., 2006; Cazzato et al., 2014, 2016). Furthermore, the limited sample size prevented us from examining differences between male and female observers and to generally explore the role of individual differences in body-related processes on the association between stillness, femininity, and aesthetic appreciation of bodies. However, in keeping with previous findings (Bernard et al., 2012, 2015), our analyses showed overlapping pattern of results in male and female participants, at least in Experiment 1 where the effects of implied motion on masculinity/femininity perception were explored. Further studies with larger sample are required to appropriately test for gender effects in body perception. Furthermore, we found overlapping results not only when data were treated at the subject level, thus aiming at generalizing at wider population of male and female observers, but also at the item level, thus aiming at generalizing the results at a wider population of male and female bodies. The use of only a limited number of variations in gender typicality (i.e., 60% vs. 90%) prevents us from describing the effect of implied motion on female and male bodies along the continuous nature of gender typicality. Future studies, thus, need to test a larger sample and use different types of stimuli (e.g., videos of real rather than computer-generated bodies in movements) with greater variations of gender typicality and greater ecological validity in order to shed light on whether the association between stillness and femininity concerns mostly perceptive mechanisms or the stereotypical meaning assigned to men and women.

## Data Availability Statement

The datasets generated for this study are available on request to the corresponding author.

## Ethics Statement

The studies involving human participants were reviewed and approved by the Comitato Etico Regionale Unico. The participants provided their written informed consent to participate in this study.

## Author Contributions

GD’A, AF, and CU conceived and designed the study. GD’A collected and analyzed the data and wrote the first draft of the manuscript. All authors revised and approved the final version of the manuscript.

## Conflict of Interest

The authors declare that the research was conducted in the absence of any commercial or financial relationships that could be construed as a potential conflict of interest.
